# A Measure of the Promiscuity of Proteins and Characteristics of Residues in the Vicinity of the Catalytic Site That Regulate Promiscuity

**DOI:** 10.1371/journal.pone.0032011

**Published:** 2012-02-16

**Authors:** Sandeep Chakraborty, Basuthkar J. Rao

**Affiliations:** Department of Biological Sciences, Tata Institute of Fundamental Research, Mumbai, India,; University of South Florida, United States of America

## Abstract

Promiscuity, the basis for the evolution of new functions through ‘tinkering’ of residues in the vicinity of the catalytic site, is yet to be quantitatively defined. We present a computational method *Prom*iscuity *I*ndice*s E*stimator (PROMISE) - based on signatures derived from the spatial and electrostatic properties of the catalytic residues, to estimate the promiscuity (PromIndex) of proteins with known active site residues and 3D structure. PromIndex reflects the number of different active site signatures that have congruent matches in close proximity of its native catalytic site, the quality of the matches and difference in the enzymatic activity. Promiscuity in proteins is observed to follow a lognormal distribution (μ = 0.28, σ = 1.1 reduced chi-square = 3.0E-5). The PROMISE predicted promiscuous functions in any protein can serve as the starting point for directed evolution experiments. PROMISE ranks carboxypeptidase A and ribonuclease A amongst the more promiscuous proteins. We have also investigated the properties of the residues in the vicinity of the catalytic site that regulates its promiscuity. Linear regression establishes a weak correlation (R^2^∼0.1) between certain properties of the residues (charge, polar, etc) in the neighborhood of the catalytic residues and PromIndex. A stronger relationship states that most proteins with high promiscuity have high percentages of charged and polar residues within a radius of 3 Å of the catalytic site, which is validated using one-tailed hypothesis tests (P-values∼0.05). Since it is known that these characteristics are key factors in catalysis, their relationship with the promiscuity index cross validates the methodology of PROMISE.

## Introduction

Jensen first proposed that promiscuity shaped the evolution of primitive cells which presumably had minimal gene content [1]–[3]. Biochemical ‘leakiness’/‘messiness’ supplemented by gene duplication ensured increased gene content and specialization [4], [5]. The catalysis of reactions distinct from the one the protein has evolved to perform, but using the same domain, is a definition of promiscuity adopted by many researchers including the current work [6]–[8]. Promiscuity is distinguished from moonlighting functions which are typically catalyzed using a domain of the protein different from the active site scaffold [9], [10].

Protein engineers tailor innovative proteins by honing an existing moonlighting or promiscuous activity [11]–[14], often without compromising its native function [15]. Inspite of the mushrooming number of promiscuous proteins being discovered, till date there is no quantitative measure of promiscuity of proteins. A recent study traces the evolution of domain promiscuity along various evolutionary pathways [16], while another method proposed a numerical analysis of promiscuous domains from genomic sequences [17]. An attempt at quantifying promiscuity provides a measure of the catalytic efficiencies of an enzyme toward a pre-defined set of substrates, but is limited in its scope and scalability [18]. Since this method assumes a uniform chemical transformation on all substrates, it is more apt for the analysis of multispecific enzymes. ‘Rigorous and quantitative measures of promiscuity’ which will measure the ‘magnitude and degree of promiscuity in a wide range of proteins’ is thus required [8].

We present a computational method for assigning a relative promiscuous index (PromIndex) to proteins with known active site and 3D structure - *Prom*iscuity *I*ndice*s E*stimator (PROMISE). PROMISE is based on the previously described method for active site detection which relies on the spatial and electrostatic properties of the catalytic residues (CLASP) [19]. Modular approaches similar to CLASP using only spatial congruence have been previously adopted [20]–[24]. Pruning based on electrostatic properties done by CLASP reduces considerable false positives compared to purely 3D matching method, as potential difference congruence implies an appropriate milieu in the catalytic site [19]. Another innovative method [25] tries to dock high-energy intermediates of various metabolites listed in a database [26] and successfully predicts the function of an unknown protein. CLASP was applied on alkaline phosphatases (APs), one of the widely studied promiscuous enzymes [27], which led to the discovery of a promiscuous protease activity in shrimp AP [19], and a promiscuous metallo-beta-lactamase activity in E. Coli AP (S. Chakraborty, R. Minda, L. Salaye, J.M. Frere, Basuthkar J. Rao, Manuscript in preparation).

A set of non-homologous proteins with known active sites and structures from the Catalytic Atlas Site (CSA) [28], “an excellent starting point for characterizing mechanistically diverse superfamilies” [29], was supplemented to include other promiscuous proteins described in a recent review [8] (Sproteins: |Sproteins| = 305).. PromIndex is now computed for each protein in the set reflecting the number of matching active site signatures from other proteins in the vicinity of the native catalytic site, the quality of the matches and how much the EC numbers differ. Since the set of proteins is unbalanced with respect to the EC numbers, random sample sets such that all Enzyme Commission (EC) numbers are equally represented are created from Sproteins. Computation of PromIndex was repeated multiple times by such random sampling to obtain statistics like mean, median and standard deviation, and PromIndex was fitted for a lognormal distribution. PROMISE ranks a carboxypeptidase A, a catechol 2,3-dioxygenase, a phosphoenolpyruvate carboxykinase, a kynureninase and a ribonuclease-A as the most promiscuous, and a ribonucleotide reductase, a lipase and a haloalkane dehalogenase as the least promiscuous proteins in Sproteins. We also establish a weak linear relationship (R^2^∼0.1) between PromIndex and certain characteristics (% of charged and polar residues) in the immediate neighborhood of the catalytic residues. Since these properties of residues are known to be intrinsic to catalysis, this relationship cross validates the methodology to quantify promiscuity. While the forward relationship is weak (i.e. high percentage of polar residues does not necessarily imply high promiscuity), the reverse relationship is quite strong (i.e. if the percentage of polar residues is low, we can say with high confidence that the protein is not very promiscuous). We establish low P-values (∼0.05) based on hypothesis testing for proportion to establish that proteins with high promiscuity mostly have high % of charged and polar residues within a radius of 3 Å from the catalytic residues, and that proteins which have low % of charged and polar residues (again within a 3 Å radius) are rarely promiscuous.

PROMISE is an automated computational method for quantifying the promiscuity of proteins, a property that till date has been described qualitatively. Promiscuity has been determined for a wide range of proteins and some of the more promiscuous proteins have been discussed in context of their known promiscuity. Computational methods [30], [31] have been used previously for the rational design of novel catalysts [32]–[34]. An added feature in PROMISE is the prediction of promiscuous functions in proteins, which requires experimental validation and often provides additional insight into the physiological role of the enzyme [35]. This feature can be easily leveraged for directed evolution, as it provides an already existing, even if incomplete, scaffold instead of depending on a de novo design [31], [36]–[38].

## Results

**Figure 1:**
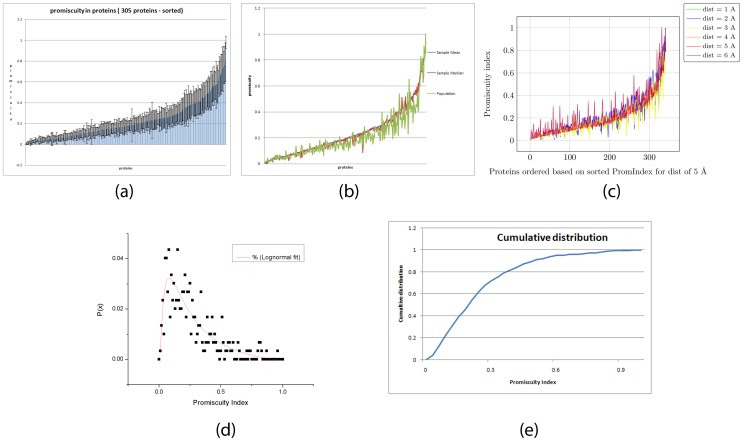
Promiscuity of proteins. (a) Mean, Standard Deviation and Median of promiscuity index (PromIndex) computed on sample sets composed of 20 proteins from each EC, and at least 30 values of PromIndex for each protein, the active site comprising of residues within a radius of 5 Å from the catalytic residues. (b) The mean from balanced sample sets with respect to EC number compared to the PromIndex computed from the full set of proteins (305 proteins). (c) PromIndex computed with radii of 1–6 Å. The proteins are sorted according to the PromIndex computed based on a 5 Å radius. (d) PromIndex was fitted for a lognormal distribution (μ = 0.28, σ = 1.1 reduced chi-square = 3.0E-5). (e) Cumulative distribution curve.

The set under consideration comprises of 305 proteins (Table S1 and Table S2). This is an unbalanced set with respect to EC representation. The number of proteins based on EC number is - EC1-47, EC2-70, EC3-101, EC4-38, EC5-26, EC6-23. We first establish that the variation in the computed promiscuity index (PromIndex) for various parameters (composition of the protein set, the radius around the catalytic residues which is considered as the active site, the weights assigned for differences in the levels in the EC numbers) is within acceptable limits. Figure 1A shows the promiscuity indices (PromIndex) computed on balanced sample sets with 20 proteins from each EC number, the active site comprising of residues within a radius of 5 Å from the catalytic residues. The proteins, plotted on the x-axis, are sorted based on the PROMISE computed promiscuity. The PromIndex of each protein has been calculated for at least 30 values, i.e. each protein figures at least 30 times in the randomly selected sets used for calculating PromIndex. Figure 1B shows the PromIndex computed on the full population (which is skewed with respect to the representation of each EC number) as compared with the mean and median obtained from balanced sets used in Figure 1A. Figure 1A and Figure 1B have the same ordering of proteins - but Figure 1A is based on (many) balanced sets and Figure 1B is based on the full set. As can be seen, the indices follow the same trend - and is not randomly scattered. At first glance it appears that this imbalance biases the promiscuity - since there are more proteins from EC 3 (hydrolases), we expect hydrolases to be more promiscuous in this computation. However, this bias can be partially negated by assigning more weightage to matches across ECs. Thus when we consider a protein from EC 6, even a single extra match with a protein from EC 3 (which are more predominant in this set), will add more to the promiscuity index neutralizing the effect of the higher number of EC 3 proteins. It is seen that the value of the weights chosen has virtually no effect on PromIndex. This is expected since the PromIndex is normalized.

Figure 1C shows the variation in PromIndex when the radius around the catalytic residues which is to be considered as part of the active site is varied from 1–6 Å. Note that the proteins are sorted according to the PromIndex as computed for a radius of 5 Å. Figure 1D shows the distribution of PromIndex for the whole population. This was fitted for a lognormal distribution
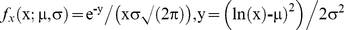
and has μ = 0.28, σ = 1.1 and reduced chi-square of 3.0E-5. Pearson's chi-squared tests the goodness of fit of an observed frequency distribution with a theoretical distribution, and a reduced chi-square statistic is the Pearson's chi-squared divided by the number of degrees of freedom. A value of the reduced chi-square close to zero suggests a good fit of the observed data with the theoretical model. Figure 1E shows the cumulative distribution for PromIndex.

**Figure 2:**
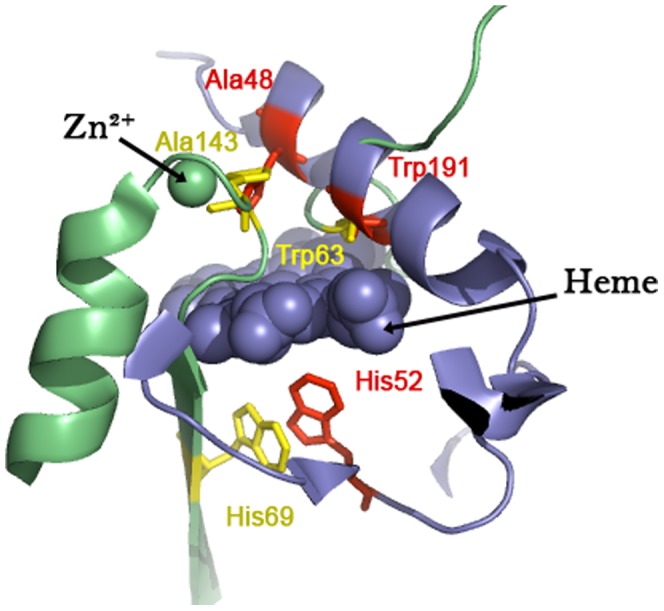
Scaffold for directed evolution. Superimposition of the predicted residues in a carboxypeptidase A (PDB id - 5CPA: in green) on a motif of active site residues from a cytochrome c peroxidase (PDB id - 1DJ1: in blue). The residues are colored in yellow and red for carboxypeptidase A and cytochrome c peroxidase respectively.

Table 1 and Table 2 list the most and least promiscuous proteins as computed by PROMISE. We touch upon some of these proteins in the discussion. These tables also enumerate some of the activities that these promiscuous proteins may have, and can serve as the starting point for directed evolution of these proteins. For example, the active site residues in a heme cytochrome c peroxidase has a good spatial and electrostatic match with residues in the active site of a Zn^2+^ carboxypeptidase A (Table 3 and Fig. 2). Assuming a heme binding site does not exist in the wild type carboxypeptidase enzyme or there is not peroxidase activity (similar to the pyruvate oxidase catalysis induced by replacing Zn^2+^ with Cu^2+^ [39]), this existing scaffold can provide a starting point for engineering heme binding sites in this protein [40].

**Table 1:** Most promiscuous proteins as computed by PROMISE: (protein with PDB id: 5CPA is the most promiscuous).

PDB	P	M	EC	L	NATIVE FUNCTION	POSSIBLE PROMISCUOUS FUNCTIONS
5CPA	1	Y	3.4.17.1	307	CARBOXYPEPTIDASE A	cytochrome c peroxidase, pyruvate oxidase, thymidine phosphorylase, hexokinase pii, chloramphenicol acetyltransferase
1MPY	0.9	Y	1.13.11.2	307	CATECHOL 2,3- DIOXYGENASE	dipeptidyl peptidase iv soluble form, purple acid phosphatase, bacterial luciferase, phosphoinositide-specific phospholipase c,
1AQ2	0.85	N	4.1.1.49	540	PHOSPHOENOLPYRUVATE CARBOXYKINASE	glutamate semialdehyde aminotransferase, endo/exocellulase e4, deoxycytidylate hydroxymethylase, aldolase carbamate carbamoylphosphate synthetase
1QZ9	0.84	Y	3.7.1.3	416	KYNURENINASE	glycine n-methyltransferase, leucyl-trna synthetase, cysteinyl-trna synthetase, citrate synthase, cytochrome p450 2b4
1EHI	0.84	Y	6.3.2.4	377	D-ALANINE:D-LACTATE LIGASE	ribonuclease alpha-sarcin, cytochrome p450 2b4, glutamate semialdehyde aminotransferase, ermc' methyltransferase, chitinase
5RSA	0.83	N	3.1.27.5	124	RIBONUCLEASE A	thymidine phosphorylase, putative biotin ligase, nadh-dependent nitrate reductase, adenylosuccinate synthetase, carboxykinase
1I9A	0.77	Y	5.3.3.2	182	ISOPENTENYL-DIPHOSPHATE DELTA-ISOMERASE	acid beta-glucosidase, ribonuclease rh, chitinase a, purine nucleoside phosphorylase, leucyl-trna synthetase
1M9C	0.76	N	5.2.1.8	165	CYCLOPHILIN A	nadh-dependent nitrate reductase, uracil phosphoribosyltransferase, cyclooxygenase-2, cytochrome c peroxidase, dehydrogenase
1ONE	0.74	Y	4.2.1.11	436	ENOLASE	purine nucleoside phosphorylase, n-ethylmaleimide sensitive factor, oxygen-insensitive nadph nitroreductase, ribonuclease alpha-sarcin, biotin ligase
1GUM	0.63	N	2.5.1.18	222	GLUTATHIONE TRANSFERASE	uracil phosphoribosyltransferase, phosphomannose isomerase, carboxykinase, tetrahydrodipicolinate n-succinyltransferase, biotin ligase

P: Promiscuity index; M: Is a metal liganded by the active site in the crystal structure; EC - Enzyme Commission number; L: Sequence length.

**Table 2:** Least promiscuous proteins as computed by PROMISE: (protein with PDB id: 3R1R is the least promiscuous).

PDB	P	M	EC	L	NATIVE FUNCTION	POSSIBLE PROMISCUOUS FUNCTIONS
3R1R	0.01	N	1.17.4.1	761	RIBONUCLEOTIDE REDUCTASE R1	glutamine phosphoribosylpyrophosphate amidotr, ferredoxin-nadp+ reductase, ribonucleoside triphosphate reductase,
1THG	0.01	N	3.1.1.3	544	LIPASE	deoxyhypusine synthase, ribonuclease t1, beta-glucuronidase, 2-enoyl-coa hydratase, ribonuclease alpha-sarcin
1B6G	0.01	N	3.8.1.5	310	HALOALKANE DEHALOGENASE	purine nucleoside phosphorylase, thymidylate synthase,
2ADM	0.02	N	2.1.1.72	421	METHYLTRANSFERASE	cytochrome p450 2b4, glutamine phosphoribosylpyrophosphate amidotr,
1POW	0.02	Y	1.2.3.3	585	PYRUVATE OXIDASE	fructose-1, 6-bisphosphatase, quinone reductase, lysozyme, adenylate kinase
1MEK	0.02	N	5.3.4.1	120	DISULFIDE ISOMERASE	(glutathione transferase a4-4), caspase-8, thymidylate synthase, ctp synthetase, glutamine phosphoribosylpyrophosphate amidotr
1T7D	0.02	N	3.4.21.89	250	SIGNAL PEPTIDASE I	udp-n-acetylmuramoyl-l-alanine/:d-glutamate l, d-dopachrome tautomerase, acetylglutamate kinase,
2CPU	0.03	N	3.2.1.1	496	ALPHA-AMYLASE	catechol 2, 3-dioxygenase, aspartyl-trna synthetase, dihy-dropteroate synthase, phospholipase a2

P: Promiscuity index; M: Is a metal liganded by the active site in the crystal structure; EC - Enzyme Commission number; L: Sequence length.

**Table 3:** Predicted residues, pairwise distances and potential differences in carboxypeptidase A and cytochrome c peroxidases using the motif (Ala48,His52,Trp191) from a cytochrome c peroxidase (PDB id: 1DJ1).

	Predicted Residues			Distances in Å			Potential differences		
	a	b	c	ab	ac	bc	ab	ac	bc
1DJ1	Ala48	His52	Trp191	6.3	11.4	13.8	329.1	317.2	−11.9
5CPA	Ala143	His69	Trp63	7.5	11.5	13.3	321.1	255.3	−65.7

Potential differences are in units of kT/e (k is Boltzmanns constant, T is the temperature in K and e is the charge of an electron).

**Figure 3:**
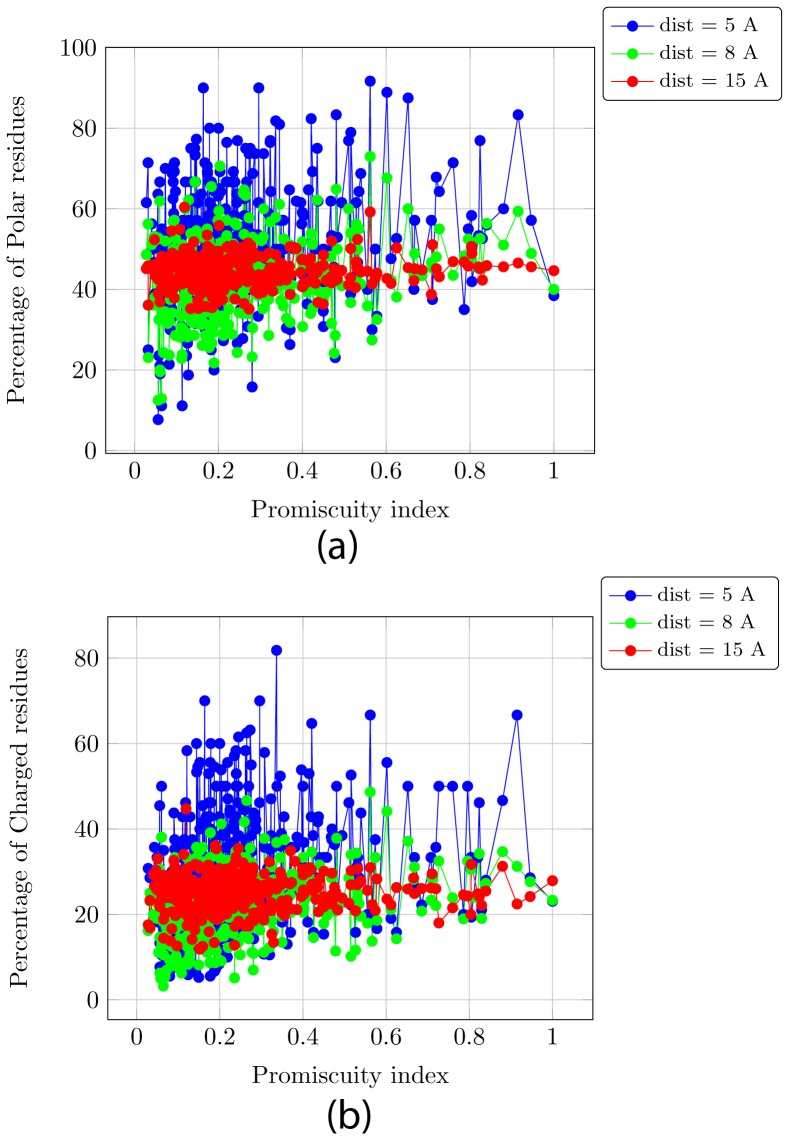
Curve fitting using Gnuplot with varying radii and various characteristics of the residues within a shell of that radius. (a) Percentage of polar residues. (b) Percentage of acidic residues. (c) Percentage of basic residues. (d) Percentage of charged (acidic and basic) residues. (e) Coefficient of determination R^2^ plotted with increasing distance from the active site.

Figure 3A and Figure 3D plots the percentage of polar and charged residues within a radial distance of 5 Å, 8 Å and 15 Å from the active residues PromIndex for each protein. The correlation between the % of charged and polar residues in the vicinity of the active site to its PromIndex is visually apparent, and this correlation is lost as we go further away from the active site. Similar correlations are observed for acidic and basic characteristics of the residues in the proximity of the active site in Figure 3B and 3C.

**Figure 4:**
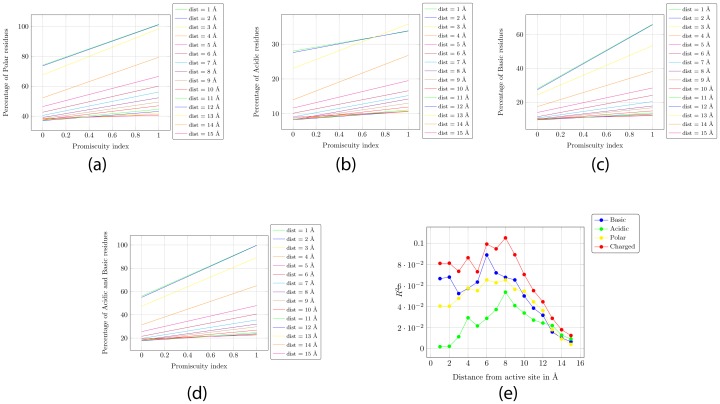
Promiscuity index plotted against the characteristics of residues within a radial distance of 5, 8 and 15 Å from the catalytic residues. (a) Percentage of polar residues. (b) Percentage of charged residues.

Figure 4 shows the linear curves fitted using Gnuplot for the data in Figure 3 for radius varying from 1 Å to 15 Å. This clearly demonstrates the gradual loss of correlation between the characteristics and PromIndex with increasing distance from the active site. Figure 4E shows the coefficient of determination (R^2^) for the fitted parameters, which establishes a weak linear correlation between the % of charged residues within 6–8 Å from the active site and PromIndex (R^2^∼0.1). Table 4 shows the decreasing variance in the percentages of polar and charged residues as the distance from the active site increases.

**Table 4:** The mean and variance in the % of polar and charged residues as the distance from the active site increases for about ∼300 proteins.

	% of Polar residues			% of Charged residues		
Distance(Å)	Mean	Median	SD	Mean	Median	SD
1	79	100	24	65	66	27
2	79	100	24	64	66	28
3	73	75	23	55	57	25
4	57	57	18	37	35	18
5	50	50	15	30	28	14
6	46	46	12	26	25	11
7	44	44	11	23	22	9
8	42	42	9	21	21	7
9	41	40	8	21	20	7
10	40	40	7	20	20	6
11	39	39	6	19	19	5
12	38	38	6	19	19	5
13	38	38	5	19	19	5
14	38	38	5	19	19	4
15	38	39	5	20	19	4

An empirical observation is that for highly promiscuous proteins the % of polar and charged residues surrounding the catalytic site is generally high. We set up a hypothesis test of this proportion to test for the statistical significance for differing radius from the catalytic site. We note from Table 4 that the mean (and median) of the percentage of polar and charged residues at a distance of 15 Å from the active site is approximately 39 and 20% respectively. Furthermore, we define highly promiscuous proteins as those having PromIndex more than the mean PromIndex of 0.28. We state that the % of promiscuous proteins (PromIndex>0.28) that has more than 39% polar residues (or 20% charged residues) is more than 80%.

Null hypothesis: P<0.80
Alternative hypothesis: P> = 0.80

**Figure 5:**
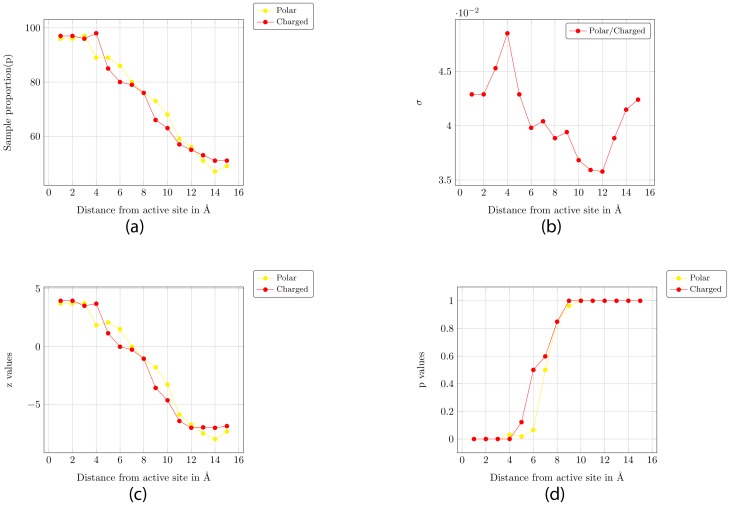
One-Tailed Hypothesis Test for proportion. The alternate hypothesis is that proteins with PromIndex >0.3, more than 80% have more than 45% of polar residues or 25% of charged residues in the vicinity of the active site. (a) Sample proportion – i.e. % of proteins with PromIndex >0.3 that have more than 45% of polar residues or 25% of charged residues in the vicinity of the active site. (b) σ value (for P = 0.8) (c) z-scores (d) P-values.

The standard deviation (σ) of the sampling distribution is given by: σ = √(P* (1−P)/n) and the test statistic (z-score) is given by z = (p−P)/σ, where p is the sample proportion. The sample proportion, the standard deviation (σ), the z-scores and the P-values are plotted in Figure 5. The P-values indicate the chance of obtaining the observed data based on the assumption that the null hypothesis is true. Since this chance is very less (less than 0.05 for distances of 3 Å around the active site), we can reject this hypothesis and claim that the alternate hypothesis is correct. Figure 5D shows that we can reject the null hypothesis for distances of 3 Å for a significance level of 0.05.

**Figure 6:**
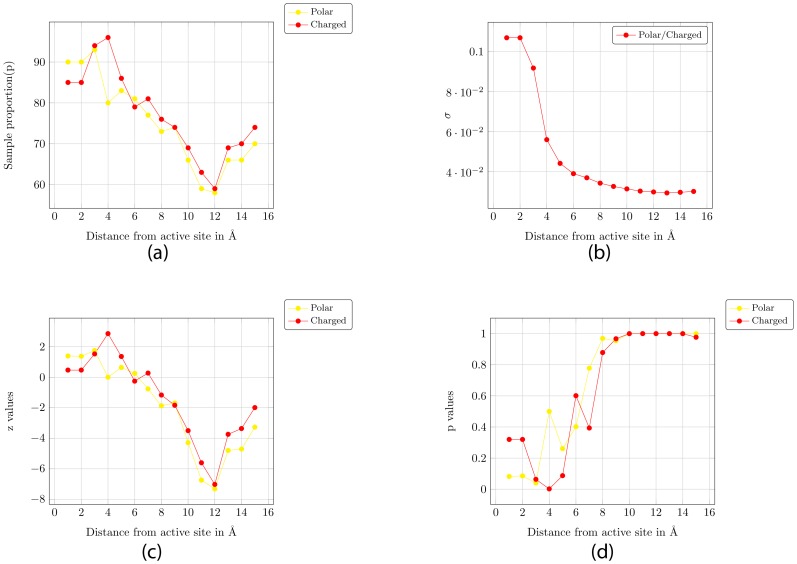
One-Tailed Hypothesis Test for proportion: The alternate hypothesis is that 80% of proteins with less than 45% of polar residues or 25% of charged residues in the vicinity of the active site have PromIndex <0.3. (a) Sample proportion – i.e. % of proteins that have less than 45% of polar residues or 25% of charged residues in the vicinity of the active site, and have PromIndex <0.3. (b) σ value (for P = 0.8) (c) z-scores (d) P-values.

Thus, we can say with a high degree of confidence that if the residues in the vicinity (3 Å) of the active site have less than 39% polar residues (or 20% charged residues) the protein is not very promiscuous. We now setup the formulation for the hypothesis test of the reverse logic - we state that if the residues in the vicinity of the active site have less than 39% polar residues (or 20% charged residues), then the probability that the protein is not promiscuous (P) is 0.80 or more. The null and alternate hypothesis is similar to the one stated above. The sample proportion, the standard deviation (σ), the z-scores and the P-values are plotted in Figure 6. Figure 6D shows that we can reject the null hypothesis for distances for a 3 Å radius around the active site for a significance level of 0.05 for both polar and charged residues.

To summarize, we show that the PromIndex is reasonably independent of the set of proteins, the radius (1–6 Å) around the catalytic residues to be considered as the active site, and the weights assigned for scoring matches across EC (Fig. 1). Next we show a weak linear relationship between certain characteristics of the residues in the active site and PromIndex (Figs. 3 and 4). Finally, using one-tailed hypothesis tests we demonstrate that most proteins with high promiscuity have high % of charged and polar residues within a distance of 3 Å from the catalytic residues (Figs. 5 and 6).

## Discussion

Promiscuity, the ability of an enzyme to catalyze multifarious activities using the same active site, was proposed by Jensen to be the basis of the evolution of complex organisms from pristine life [1]. Primitive life presumably had minimal gene content and a limited arsenal of enzymes. Subsequent gene duplication and ‘tinkering’ of ‘plasticity’ residues in the proximity of the active site honed a few select activities into ‘specialist’ enzymes [4], [8], [41]–[43]. Regardless, the remnants of the secondary activities under neutral drift [44] retained the potential to resurface under changing selection pressures [45], [46]. Modern biotechnology has exploited these latent capabilities to conjure new proteins under laboratory conditions [12], and innovate drugs that manipulate such ‘messiness’ [5], [47].

Inspite of the intense efforts being devoted to gain insights into promiscuity, currently there is no formal method to quantify and correlate promiscuity of proteins. A recently proposed method for measuring catalytic efficiencies of an enzyme toward a pre-defined set of substrates lacks scalability, and is more applicable to substrate promiscuity [18]. We propose an automated computational methodology for computing the relative promiscuity of a set of proteins with known active sites and structure -Promiscuity Indices Estimator (PROMISE) -based on the spatial and electrostatic properties of the catalytic residues. Electrostatic interactions determine various properties of biomolecules such as catalytic activity, ligand binding, structure and stability [48]. Finite difference Poisson-Boltzmann electrostatics is used to compute potential differences [49], [50]. Although, the CLASP signature for any function comprises of a few residues, it implicitly encodes the surroundings. Potential difference congruence implies a conducive milieu for the particular function (hydrophobicity, polarity, presence in a cleft, etc). Random sampling from a set of proteins with known active site and structure, primarily culled from CSA [28], establishes statistics like mean, median and standard deviation (Fig. 1). The promiscuity index for the population is fitted for a lognormal distribution. Such skewed distributions typically result from stochastic effects that move a variable towards more probable states leading to multiplicative variations about the mean [51]. The CSA database can also be supplemented by methods that automatically generate structural motifs [52], [53].

We now discuss a few of the more promiscuous proteins, as computed by PROMISE (Table 1). Carboxypeptidase A (PDB id: 5CPA) has been predicted to be the most promiscuous amongst the proteins analyzed. It was demonstrated in 1976 that replacing the metal ion introduced oxidase catalysis properties in this protein, legitimatizing the PROMISE prediction that this protein might have cytochrome c peroxidase and pyruvate oxidase activities [39]. The next protein, a dioxygenase (PDB id: 1MPY), has been known to hydrolyze esters [54]. PROMISE predicts that this dioxygenase might have purple acid phosphatase, an esterase activity. Carboxykinases (PDB id: 1AQ2) are assumed to be very ancient proteins since they are ubiquitous in the three domains of life. Phylogenomic analysis indicates early evolution of the carboxylase family [55], substantiating Jensen's hypothesis that the pristine proteins were very promiscuous [1]. Ribonuclease A (PDB id: 5RSA) is another example of an ancient multifaceted protein, that has been honed by evolution to maximum efficiency [56]. However triosephosphate isomerase (PDB id: 1HTI), another diffusion controlled enzyme, is predicted to be mildly promiscuous (index of 0.2) [57]. This appears to be a more logical trade-off between efficiency and promiscuity. Most enzymes are moderately efficient retaining the potential to adapt to physiochemical environmental constraints [14], [58]. The trade-off between thermodynamic stability, silent mutations and the ability to acquire new functions are well recognized [59]–[61]. The uniqueness of Ribonuclease A is the ability to have perfect catalytic efficiency, attain thermodynamic stability and still possess the promiscuity to adapt. A protein from the enolase superfamily (PDB id: 1ONE) is also predicted to be highly promiscuous. The mechanistically diverse enolase superfamily is known to catalyze numerous catalytic reactions, most of which share a partial reaction [29], [62]. Table 2 shows some of the least promiscuous proteins as computed by PROMISE. It is best to reiterate here that substrate promiscuity as demonstrated by haloalkane dehalogenase (PDB id: 1B6G) does not add to the promiscuity index computed by PROMISE, since the protein set is non-homologous.

Table 1 and Table 2 raise a pertinent question - what makes a protein promiscuous? [63]. It can be seen that while sequence length and the kind of activity (EC number) it performs has no bearing on the promiscuity of a protein, most promiscuous proteins are seen to be metal dependent whereas the least promiscuous are typically not. Metal coordination of nucleophilic groups has been known to enhance the catalytic repertoire of metalloenzymes. Also, we see little correlation between promiscuity functions and EC numbers. It has been previously stated that folds and primary EC numbers are unrelated [64], [65]. We demonstrate a weak linear relationship between some features of the residues in the vicinity (about 6–8 Å) of the catalytic site (Fig. 4E). Furthermore, hypothesis test on proportions establishes with low P-values (∼0.05) that proteins with high promiscuity mostly have high % of charged and polar residues, and that proteins which have low % of charged and polar residues are rarely promiscuous within a radius of 3 Å from the catalytic residues. Note that we do not state that high % of charged and polar residues implies high promiscuity - thus, there is no straight forward method to make a protein more promiscuous. However, if we mutate the charged and polar residues in the vicinity of the catalytic residues of a promiscuous protein to uncharged and non-polar residues respectively, then we state and demonstrate that the protein will become less promiscuous.

PROMISE quantifies the promiscuity of proteins, a property hitherto qualitatively described. An additional feature of PROMISE is the prediction of promiscuous activities in proteins. This feature can be leveraged to obtain starting points for directed evolution by protein engineers interested in bestowing a protein with a non-existent or weak function.

## Materials and Methods

### 4.1 Algorithm

The CLASP algorithm has been detailed previously [19]. In summary, given the active site residues from a protein with known structure a signature encapsulating the spatial and electrostatic properties of the catalytic site is used to search for congruent matches in a query protein, generating a score which reflects the likelihood that the activity in the reference protein exists in the query protein. Figure S1 details the PROMISE methodology to compute the promiscuity index (PromIndex) of proteins. We start with a non-homologous set of proteins with known active site and structure while ensuring that equal numbers of proteins (n) from each EC number are present in the set. The active site residues are used to generate 6n signatures, and CLASP scores are generated for each signature and each protein: Scores ⇐ {Scores_ij_ : µi, j ∈ 6n, i≠j}. Lower CLASP scores denote better congruence; hence we discard all matches whose scores are more than a user defined threshold (thr). Another user defined option (shellrad) determines the residues which are to be considered in the vicinity of the active site (*Vic*_i_). This is the radius around each catalytic residue that is considered to be a part of the ‘catalytic site’. Consider the case when CLASP predicts that protein P_i_ has the activity seen in P_i_ - PromIndex is incremented based on the differences in levels of the EC numbers of P_i_ and P_j_ and an empirical score (10 for e.g.) if and only if the predicted active site (Pred_ij_) overlaps with the (*Vic*_i_) residues, For example, 4.3.2 and 4.3.1 would differ in level 3, and the score increment would be 10/3, while 4.3.2 and 3.3.2 would differ in level 1 and the score would be incremented by 10/1. PromIndex is now adjusted based on the CLASP scores - a lower score implies a higher probability that the function exists and results in a better promiscuity score. Finally, these indices are normalized - the most promiscuous protein has a promiscuity index of 1.

### 4.2 Tools

Adaptive Poisson-Boltzmann Solver [50] (APBS) and the PDB2PQR package [66] package was used to calculate the potential difference between the reactive atoms of the corresponding proteins. The APBS parameters are set as follows - solute dielectric: 2, solvent dielectric: 78, solvent probe radius: 1.4 Å, Temperature: 298 K and 0 ionic strength. APBS writes out the electrostatic potential in dimensionless units of kT/e where k is Boltzmann's constant, T is the temperature in K and e is the charge of an electron. The ‘pepstats’ program from the Emboss suite of tools was used to obtain statistics of protein properties [67]. We extensively integrated and used the freely available BioPerl [68] modules. Origin was used for curve fitting. Statistics::Distributions package from CPAN was used for obtaining p-values.

### 4.3 Dataset selection

The Catalytic Site Atlas (CSA), available online, provides catalytic residue annotation for enzymes in the PDB [28]. The database consists of two types of annotated sites: an original hand-annotated set containing information extracted from the primary literature and a homologous set containing residues inferred by PSIBLAST [69]. We downloaded the file CSA 2 2 12.list from the CSA site and extracted about ∼300 proteins where the active site residues were extracted from literature, had either 3–6 residues specified in the active site and were all confined to one polypeptide. We supplemented the hand annotated set provided by CSA to include other promiscuous proteins described in a recent review [8], and other proteins of interest which were not in the CSA database (Table S1 and Table S2). There were 305 proteins in all. The number of proteins based on EC number is - EC1-47, EC2-70, EC3-101, EC4-38, EC5-26, EC6-23.

## Supporting Information

Figure S1 **Algorithm for generating Promiscuity Index.**
(PDF)

Table S1 **Set of non-homologous proteins with known active sites.**
(PDF)

Table S2 **Proteins added to the CSA list to include some proteins of interest.**
(PDF)
