# Supplementary material for: A Measure of the Promiscuity of Proteins and Characteristics of Residues in the Vicinity of the Catalytic Site That Regulate Promiscuity
Source: PLoS One. 2012 Feb 16;7(2):e32011. doi: 10.1371/journal.pone.0032011 (PMC3281107; doi:10.1371/journal.pone.0032011)
Supplement: Figure S1 — Algorithm for generating Promiscuity Index. (PDF) [file pone.0032011.s001.pdf]

Algorithm 1: PROMISE - Prom*iscuity* I*ndice*s E*stimator*

Input: $6n \Leftarrow$ number of proteins in the set - each EC number has n elements
Input: $Score \Leftarrow \{Score_{ij} : \forall i, j \in 6n, i \neq j\}$ CLASP score when motif from Protein j is queried in Protein i
Input: $Pred \Leftarrow \{Pred_{ij} : \forall i, j \in 6n, i \neq j\}$ Predicted residues in Protein i responsible for activity known for Protein j
Input: $EC \Leftarrow \{EC_{ij} : \forall i, j \in 6n, i \neq j\}$ Level in which the EC numbers of Protein i and Protein j differ
Input: $thr \Leftarrow$ Ignore matches whose CLASP score $Score_{ij}$ is less than this value
Input: $shellrad \Leftarrow$ Radius of shell which is to be considered as the active site - 5Å
Input: $Vic \Leftarrow \{Vic_i : \forall i \in 6n\}$ Residues in vicinity of active site of Protein i within distance $shellrad$
Output: $PromIdx \Leftarrow \{PromIdx_i : \forall i \in 6n\}$ Promiscuity Indices

```
begin
    for i ← 1 to 6n do
        PromIdx_i = 0; empirical = 10 ; // initialize
        for j ← 1 to 6n, i ≠ j do
            /* Discard poor matches*/
            if(Score_ij ≥ thr) continue ;

            /* Score of difference in catalytic activity - ie level in
            which the EC numbers of the native and predicted activity differ*/
            δ_scoreij = empirical / EC_ij ;

            /* Score for the quality of the spatial and
            electrostatic congruence of the predicted function*/
            δ_scoreij = δ_scoreij / Score_ij ;

            /* check if in vicinity of the native active site */
            if(Pred_ij ∧ Vic_ij ≠ φ ) then
            PromIdx_i = PromIdx_i + δ_scoreij ;
            endif
        end
    end
    Normalize(PromIdx); // most promiscuous protein has a promiscuity index of 1
end
```
