# Supplementary material for: A Measure of the Promiscuity of Proteins and Characteristics of Residues in the Vicinity of the Catalytic Site That Regulate Promiscuity
Source: PLoS One. 2012 Feb 16;7(2):e32011. doi: 10.1371/journal.pone.0032011 (PMC3281107; doi:10.1371/journal.pone.0032011)
Supplement: Table S1 — Set of non-homologous proteins with known active sites. (PDF) [file pone.0032011.s002.pdf]

Supplementary Table 1: Set of non-homologous proteins with known active sites

PDB	EC number	Native function
1FJO	3.4.24.27	THERMOLYSIN
1PO5	1.14.14.1	Cytochrome P450 2B4
1GUM	2.5.1.18	PROTEIN (GLUTATHIONE TRANSFERASE A4-4)
1DBT	4.1.1.23	OROTIDINE 5'-PHOSPHATE DECARBOXYLASE
2GVW	3.1.8.2	Phosphotriesterase
1ONE	4.2.1.11	ENOLASE
1V04	3.1.1.2	SERUM PARAOXONASE/ARYLESTERASE 1
1DPT	4.1.1.84	D-DOPACHROME TAUTOMERASE
1QRG	4.2.1.1	CARBONIC ANHYDRASE
1HDH	3.1.6.1	ARYLSULFATASE
1CA1	3.1.4.3	ALPHA-TOXIN
1A0J	3.4.21.4	TRYPSIN
1BTL	3.5.2.6	BETA-LACTAMASE TEM1
1ZNB	3.5.2.6	METALLO-BETA-LACTAMASE
1A4L	3.5.4.4	ADENOSINE DEAMINASE
1PTD	4.6.1.13	PHOSPHATIDYLINOSITOL-SPECIFIC PHOSPHOLIPASE C
1A79	3.1.27.9	TRNA ENDONUCLEASE
1A82	6.3.3.3	DETHIOBIOTIN SYNTHETASE
1A8H	6.1.1.10	METHIONYL-TRNA SYNTHETASE
1A95	2.4.2.22	XANTHINE-GUANINE PHOSPHORIBOSYLTRANSFERASE
1AAM	2.6.1.1	ASPARTATE AMINOTRANSFERASE
1AB4	5.99.1.3	GYRASE A
1AFW	2.3.1.16	3-KETOACETYL-COA THIOLASE
1AGY	3.1.1.74	CUTINASE
1AJ0	2.5.1.15	DIHYDROPTEROATE SYNTHASE
1AJ8	2.3.3.1	CITRATE SYNTHASE
1AKM	2.1.3.3	ORNITHINE TRANSCARBAMYLASE
1AKO	3.1.11.2	EXONUCLEASE III
1AL6	2.3.3.1	CITRATE SYNTHASE
1ALD	4.1.2.13	ALDOLASE A
1AM2	5.99.1.3	MXE GYRA INTEIN
1AQ0	3.2.1.73	1,3-1,4-BETA-GLUCANASE
1AQ2	4.1.1.49	PHOSPHOENOLPYRUVATE CARBOXYKINASE
1ASY	6.1.1.12	ASPARTYL-tRNA SYNTHETASE
1AUO	3.1.1.1	CARBOXYLESTERASE
1AZW	3.4.11.5	PROLINE IMINOPEPTIDASE
1AZY	2.4.2.4	THYMIDINE PHOSPHORYLASE
1B2M	3.1.27.3	RIBONUCLEASE T1
1B5D	2.1.2.8	PROTEIN (DEOXYCYTIDYLATE HYDROXYMETHYLASE)
1B6B	2.3.1.87	PROTEIN (ARYLALKYLAMINE N-ACETYLTRANSFERASE)
1B6G	3.8.1.5	HALOALKANE DEHALOGENASE
1B6T	2.7.7.3	PROTEIN (PHOSPHOPANTETHEINE ADENYLYLTRANSFERA
1B73	5.1.1.3	GLUTAMATE RACEMASE
1B8G	4.4.1.14	PROTEIN (1-AMINOCYCLOPROPANE-1-CARBOXYLATE SY
1B93	4.2.3.3	PROTEIN (METHYLGLYOXAL SYNTHASE)
1BD3	2.4.2.9	URACIL PHOSPHORIBOSYLTRANSFERASE

Continued on Next Page...

Supplementary Table 1 – Continued

PDB	EC number	Native function
1BF2	3.2.1.68	ISOAMYLASE
1BFD	4.1.1.7	BENZOYLFORMATE DECARBOXYLASE
1BG0	2.7.3.3	ARGININE KINASE
1BHG	3.2.1.31	BETA-GLUCURONIDASE
1BIB	6.3.4.15	BIR A
1BIX	4.2.99.18	AP ENDONUCLEASE 1
1BJO	2.6.1.52	PHOSPHOSERINE AMINOTRANSFERASE
1BMT	2.1.1.13	METHIONINE SYNTHASE
1BOL	3.1.27.1	PROTEIN (RIBONUCLEASE RH)
1BOO	2.1.1.113	PROTEIN (N-4 CYTOSINE-SPECIFIC METHYLTRANSFER
1BP2	3.1.1.4	PHOSPHOLIPASE A2
1BS0	2.3.1.47	PROTEIN (8-AMINO-7-OXONANOATE SYNTHASE)
1BS4	3.5.1.88	PROTEIN (PEPTIDE DEFORMYLASE)
1BS9	3.1.1.72	ACETYL XYLAN ESTERASE
1BVZ	3.2.1.135	PROTEIN (ALPHA-AMYLASE II)
1BWD	2.1.4.2	PROTEIN (INOSAMINE-PHOSPHATE AMIDINOTRANSFERA
1BWP	3.1.1.47	PLATELET-ACTIVATING FACTOR ACETYLHYDROLASE
1BWZ	5.1.1.7	PROTEIN (DIAMINOPIMELATE EPIMERASE)
1BZC	3.1.3.48	PROTEIN (PROTEIN-TYROSINE-PHOSPHATASE)
1BZY	2.4.2.8	HYPOXANTHINE-GUANINE PHOSPHORIBOSYLTRANSFERAS
1C2T	2.1.2.2	GLYCINAMIDE RIBONUCLEOTIDE TRANSFORMYLASE
1C82	4.2.2.1	HYALURONATE LYASE
1CBG	3.2.1.21	CYANOGENIC BETA-GLUCOSIDASE
1CD5	3.5.99.6	PROTEIN (GLUCOSAMINE 6-PHOSPHATE DEAMINASE)
1CDG	2.4.1.19	CYCLODEXTRIN GLYCOSYL-TRANSFERASE
1CEL	3.2.1.91	1,4-BETA-D-GLUCAN CELLOBIOHYDROLASE I
1CG2	3.4.17.11	CARBOXYPEPTIDASE G2
1CGK	2.3.1.74	PROTEIN (CHALCONE SYNTHASE)
1CHM	3.5.3.3	CREATINE AMIDINOHYDROLASE
1CMX	3.4.19.12	PROTEIN (UBIQUITIN YUH1-UBAL)
1CNS	3.2.1.14	CHITINASE
1CTN	3.2.1.14	CHITINASE A
1CVR	3.4.22.37	GINGIPAIN R
1CWY	2.4.1.25	AMYLOMALTASE
1CZF	3.2.1.15	POLYGALACTURONASE II
1D2H	2.1.1.20	GLYCINE N-METHYLTRANSFERASE
1D6M	5.99.1.2	DNA TOPOISOMERASE III
1D7R	4.1.1.64	PROTEIN (2,2-DIALKYLGLYCINE DECARBOXYLASE (PY
1D8C	2.3.3.9	MALATE SYNTHASE G
1D8H	3.1.3.33	mRNA TRIPHOSPHATASE CET1
1DAA	2.6.1.21	D-AMINO ACID AMINOTRANSFERASE
1DB3	4.2.1.47	GDP-MANNOSE 4,6-DEHYDRATASE
1DD8	2.3.1.41	BETA-KETOACYL [ACYL CARRIER PROTEIN] SYNTHASE
1DE3	3.1.27.10	RIBONUCLEASE ALPHA-SARCIN
1DE6	5.3.1.14	L-RHAMNOSE ISOMERASE
1DFO	2.1.2.1	SERINE HYDROXYMETHYLTRANSFERASE
1DI1	4.2.3.9	ARISTOLOCHENE SYNTHASE

Continued on Next Page…

Supplementary Table 1 – Continued

PDB	EC number	Native function
1DIZ	3.2.2.21	3-METHYLADENINE DNA GLYCOSYLASE II
1DJ1	1.11.1.5	CYTOCHROME C PEROXIDASE
1DL2	3.2.1.113	CLASS I ALPHA-1,2-MANNOSIDASE
1DNK	3.1.21.1	PROTEIN (DEOXYRIBONUCLEASE I (DNASE I) (E.C.3
1DNP	4.1.99.3	DNA PHOTOLYASE
1DUB	4.2.1.17	2-ENOYL-COA HYDRATASE
1E19	2.7.2.2	CARBAMATE KINASE-LIKE CARBAMOYLPHOSPHATE SYNT
1E1O	6.1.1.6	LYSYL-TRNA SYNTHETASE
1E2T	2.3.1.118	N-HYDROXYARYLAMINE O-ACETYLTRANSFERASE
1E3V	5.3.3.1	STEROID DELTA-ISOMERASE
1EC9	4.2.1.40	GLUCARATE DEHYDRATASE
1ECF	2.4.2.14	GLUTAMINE PHOSPHORIBOSYLPYROPHOSPHATE AMIDOTR
1EHI	6.3.2.4	D-ALANINE:D-LACTATE LIGASE
1EI5	3.4.11.19	D-AMINOPEPTIDASE
1EIX	4.1.1.23	OROTIDINE 5'-MONOPHOSPHATE DECARBOXYLASE
1ELS	4.2.1.11	ENOLASE
1EQ2	5.1.3.20	ADP-L-GLYCERO-D-MANNOHEPTOSE 6-EPIMERASE
1EUQ	6.1.1.18	GLUTAMINYL-TRNA SYNTHETASE
1EUU	3.2.1.18	SIALIDASE
1EYI	3.1.3.11	FRUCTOSE-1,6-BISPHOSPHATASE
1EYP	5.5.1.6	CHALCONE-FLAVONONE ISOMERASE 1
1F2D	3.5.99.7	1-AMINOCYCLOPROPANE-1-CARBOXYLATE DEAMINASE
1F6D	5.1.3.14	UDP-N-ACETYLGLUCOSAMINE 2-EPIMERASE
1F75	2.5.1.31	UNDECAPRENYL PYROPHOSPHATE SYNTHETASE
1F8M	4.1.3.1	ISOCITRATE LYASE
1F8X	2.4.2.6	NUCLEOSIDE 2-DEOXYRIBOSYLTRANSFERASE
1FCQ	3.2.1.35	HYALURONOGLUCOSAMINIDASE
1FGH	4.2.1.3	ACONITASE
1FHL	3.2.1.89	BETA-1,4-GALACTANASE
1FO6	3.5.1.77	N-CARBAMoYL-D-AMINO-ACID AMIDOHYDROLASE
1FY2	3.4.13.21	ASPARTYL DIPEPTIDASE
1G0D	2.3.2.13	PROTEIN-GLUTAMINE GAMMA-GLUTAMYLTRANSFERASE
1G6T	2.5.1.19	EPSP SYNTHASE
1G99	2.7.2.1	ACETATE KINASE
1GCB	3.4.22.40	GAL6 HG (EMTS) DERIVATIVE
1GEQ	4.2.1.20	TRYPTOPHAN SYNTHASE ALPHA-SUBUNIT
1GIM	6.3.4.4	ADENYLOSUCCINATE SYNTHETASE
1GLO	3.4.22.27	CATHEPSIN S
1GLV	6.3.2.3	GLUTATHIONE SYNTHASE
1GPA	2.4.1.1	GLYCOGEN PHOSPHORYLASE A
1GPM	6.3.5.2	GMP SYNTHETASE
1GPR	2.7.1.69	GLUCOSE PERMEASE
1GQ8	3.1.1.11	PECTIN METHYLESTERASE
1H7O	4.2.1.24	5-AMINOLAEVULINIC ACID DEHYDRATASE
1HPL	3.1.1.3	LIPASE
1HRK	4.99.1.1	FERROCHELATASE
1HTI	5.3.1.1	TRIOSEPHOSPHATE ISOMERASE

Continued on Next Page...

Supplementary Table 1 – Continued

PDB	EC number	Native function
1HY3	2.8.2.4	ESTROGEN SULFOTRANSFERASE
1I8T	5.4.99.9	UDP-GALACTOPYRANOSE MUTASE
1I9A	5.3.3.2	ISOPENTENYL-DIPHOSPHATE DELTA-ISOMERASE
1IG8	2.7.1.1	hexokinase PII
1ILE	6.1.1.5	ISOLEUCYL-TRNA SYNTHETASE
1IU4	2.3.2.13	microbial transglutaminase
1JS4	3.2.1.4	ENDO/EXOCELLULASE E4
1K4L	4.1.99.12	3,4-Dihydroxy-2-Butanone 4-Phosphate Synthase
1KAS	2.3.1.179	BETA-KETOACYL ACP SYNTHASE II
1KEZ	2.3.1.94	ERYTHRONOLIDE SYNTHASE
1KZL	2.5.1.9	Riboflavin Synthase
1LCB	2.1.1.45	THYMIDYLATE SYNTHASE
1LI5	6.1.1.16	CYSTEINYL-TRNA SYNTHETASE
1LJL	3.1.3.48	arsenate reductase
1LTQ	2.7.1.78	POLYNUCLEOTIDE KINASE
1M0T	6.3.2.3	glutathione synthetase
1M9C	5.2.1.8	Cyclophilin A
1MDR	5.1.2.2	MANDELATE RACEMASE
1MEK	5.3.4.1	PROTEIN DISULFIDE ISOMERASE
1MEN	2.1.2.2	Phosphoribosylglycinamide formyltransferase
1MLA	2.3.1.39	MALONYL-COENZYME A ACYL CARRIER PROTEIN TRANS
1MOQ	2.6.1.16	GLUCOSAMINE 6-PHOSPHATE SYNTHASE
1NBA	3.5.1.59	N-CARBAMOYLSARCOSINE AMIDOHYDROLASE
1NBF	3.1.2.15	Ubiquitin carboxyl-terminal hydrolase 7
1NKK	3.4.21.97	Capsid protein P40
1NLN	3.4.22.39	Adenain
1NLU	3.4.21.100	SEDOLISIN
1NMW	5.2.1.8	Peptidyl-prolyl cis-trans isomerase NIMA-inte
1NSF	3.6.4.6	N-ETHYLMALEIMIDE SENSITIVE FACTOR
1NU3	3.3.2.8	limonene-1,2-epoxide hydrolase
1OBA	3.2.1.17	LYSOZYME
1OG1	2.4.2.31	T-CELL ECTO-ADP-RIBOSYLTRANSFERASE 2
1OH9	2.7.2.8	ACETYLGLUTAMATE KINASE
1ONR	2.2.1.2	TRANSALDOLASE B
1ORD	4.1.1.17	ORNITHINE DECARBOXYLASE
1OTG	5.3.3.10	5-CARBOXYMETHYL-2-HYDROXYMUCONATE ISOMERASE
1OYG	2.4.1.10	levansucrase
1P1X	4.1.2.4	Deoxyribose-phosphate aldolase
1PFK	2.7.1.11	PHOSPHOFRUCTOKINASE
1PFQ	3.4.14.5	Dipeptidyl peptidase IV soluble form
1PJH	5.3.3.8	enoyl-CoA isomerase; Eci1p
1PKN	2.7.1.40	PYRUVATE KINASE
1PMI	5.3.1.8	PHOSPHOMANNOSE ISOMERASE
1PVD	4.1.1.1	PYRUVATE DECARBOXYLASE
1PYM	5.4.2.9	PROTEIN (PHOSPHOENOLPYRUVATE MUTASE)
1QAM	2.1.1.48	ERMC' METHYLTRANSFERASE
1QFE	4.2.1.10	PROTEIN (3-DEHYDROQUINATE DEHYDRATASE)

Continued on Next Page…

Supplementary Table 1 – Continued

PDB	EC number	Native function
1QFM	3.4.21.26	PROTEIN (PROLYL OLIGOPEPTIDASE)
1QGX	3.1.3.7	3',5'-ADENOSINE BISPHOSPHATASE
1QHF	5.4.2.1	PROTEIN (PHOSPHOGLYCERATE MUTASE)
1QHO	3.2.1.133	ALPHA-AMYLASE
1QJ4	4.1.2.37	HYDROXYNITRILE LYASE
1QK2	3.2.1.91	CELLOBIOHYDROLASE CEL6A (FORMERLY CALLED CBH
1QQ5	3.8.1.2	PROTEIN (L-2-HALOACID DEHALOGENASE)
1QRR	3.13.1.1	sulfolipid biosynthesis (SQD1) PROTEIN
1QTN	3.4.22.61	CASPASE-8
1QX3	3.4.22.56	Apopain
1QZ9	3.7.1.3	KYNURENINASE
1RBA	4.1.1.39	RUBISCO
1RK2	2.7.1.15	RIBOKINASE
1ROZ	2.5.1.46	Deoxyhypusine synthase
1RPT	3.1.3.2	PROSTATIC ACID PHOSPHATASE
1RPX	5.1.3.1	PROTEIN (RIBULOSE-PHOSPHATE 3-EPIMERASE)
1S95	3.1.3.16	Serine/threonine protein phosphatase 5
1SCA	3.4.21.62	SUBTILISIN CARLSBERG
1SES	6.1.1.11	SERYL-tRNA SYNTHETASE
1SLL	4.2.2.15	SIALIDASE L
1SMN	3.1.30.2	EXTRACELLULAR ENDONUCLEASE
1SNN	4.1.99.12	3,4-dihydroxy-2-butanone 4-phosphate synthase
1SSX	3.4.21.12	Alpha-lytic protease
1STD	4.2.1.94	SCYTALONE DEHYDRATASE
1T7D	3.4.21.89	Signal peptidase I
1THG	3.1.1.3	LIPASE
1UAE	2.5.1.7	UDP-N-ACETYLGLUCOSAMINE ENOLPYRUVYL TRANSFERA
1UAG	6.3.2.9	UDP-N-ACETYLMURAMOYL-L-ALANINE/:D-GLUTAMATE L
1ULA	2.4.2.1	PURINE NUCLEOSIDE PHOSPHORYLASE
1UOK	3.2.1.10	OLIGO-1,6-GLUCOSIDASE
1VAS	3.1.25.1	PROTEIN (T4 ENDONUCLEASE V (E.C.3.1.25.1))
1VBN	6.1.1.1	Tyrosyl-tRNA synthetase
1VCM	6.3.4.2	CTP synthetase
1VR7	4.1.1.50	S-adenosylmethionine decarboxylase proenzyme
1W93	6.4.1.2	ACETYL-COENZYME A CARBOXYLASE
1WD8	3.5.3.15	Protein-arginine deiminase type IV
1WKB	6.1.1.4	Leucyl-tRNA synthetase
1XTC	2.4.2.36	CHOLERA TOXIN
1XYZ	3.2.1.8	1,4-BETA-D-XYLAN-XYLANOHYDROLASE
1YSC	3.4.16.5	SERINE CARBOXYPEPTIDASE
1YTW	3.1.3.48	YERSINIA PROTEIN TYROSINE PHOSPHATASE
1Z9H	5.3.99.3	membrane-associated prostaglandin E synthase-
1ZIO	2.7.4.3	ADENYLATE KINASE
1ZRZ	2.7.11.13	Protein kinase C, iota
2ACE	3.1.1.7	ACETYLCHOLINESTERASE
2ADM	2.1.1.72	ADENINE-N6-DNA-METHYLTRANSFERASE TAQI
2AMG	3.2.1.60	1,4-ALPHA-D-GLUCAN MALTOTETRAHYDROLASE

Continued on Next Page...

Supplementary Table 1 – Continued

PDB	EC number	Native function
2AYH	3.2.1.73	1,3-1,4-BETA-D-GLUCAN 4-GLUCANOHYDROLASE
2BVC	6.3.1.2	GLUTAMINE SYNTHETASE 1
2CPU	3.2.1.1	ALPHA-AMYLASE
2D3A	6.3.1.2	glutamine synthetase
2EJ9	6.3.4.15	Putative biotin ligase
2F61	3.2.1.45	Acid beta-glucosidase
2GSA	5.4.3.8	GLUTAMATE SEMIALDEHYDE AMINOTRANSFERASE
2ISD	3.1.4.11	PHOSPHOINOSITIDE-SPECIFIC PHOSPHOLIPASE C, IS
2LIP	3.1.1.3	LIPASE
2NMT	2.3.1.97	MYRISTOYL-COA:PROTEIN N-MYRISTOYLTRANSFERASE
2OAT	2.6.1.13	ORNITHINE AMINOTRANSFERASE
2PFL	2.3.1.54	PROTEIN (PYRUVATE FORMATE-LYASE)
2PTH	3.1.1.29	PEPTIDYL-TRNA HYDROLASE
2QMO	6.3.3.3	Dethiobiotin synthetase
2TDT	2.3.1.117	TETRAHYDRODIPICOLINATE N-SUCCINYLTRANSFERASE
2YPN	2.5.1.61	PROTEIN (HYDROXYMETHYLBILANE SYNTHASE)
3CLA	2.3.1.28	TYPE III CHLORAMPHENICOL ACETYLTRANSFERASE
3CSM	5.4.99.5	CHORISMATE MUTASE
3PVA	3.5.1.11	PROTEIN (PENICILLIN V ACYLASE)
4KBP	3.1.3.2	PURPLE ACID PHOSPHATASE
5CPA	3.4.17.1	CARBOXYPEPTIDASE A
5EAT	4.2.3.9	5-EPI-ARISTOLOCHENE SYNTHASE
5FIT	3.6.1.29	FRAGILE HISTIDINE TRIAD PROTEIN
5RSA	3.1.27.5	RIBONUCLEASE A
7ODC	4.1.1.17	PROTEIN (ORNITHINE DECARBOXYLASE)
8PCH	3.4.22.16	CATHEPSIN H
1A8S	1.11.1.10	CHLOROPEROXIDASE F
1AFR	1.14.19.2	DELTA9 STEAROYL-ACYL CARRIER PROTEIN DESATURA
1APX	1.11.1.11	CYTOSOLIC ASCORBATE PEROXIDASE
1BRM	1.2.1.11	ASPARTATE-SEMIALDEHYDE DEHYDROGENASE
1BWL	1.6.99.1	PROTEIN (NADPH DEHYDROGENASE 1)
1COY	1.1.3.6	CHOLESTEROL OXIDASE
1D3G	1.3.3.1	DIHYDROOROTATE DEHYDROGENASE
1D4A	1.6.5.2	QUINONE REDUCTASE
1D4C	1.3.99.1	FLAVOCYTOCHROME C FUMARATE REDUCTASE
1DII	1.17.99.1	P-CRESOL METHYLHYDROXYLASE
1DJL	1.6.1.2	TRANSHYDROGENASE DIII
1DLI	1.1.1.22	UDP-GLUCOSE DEHYDROGENASE
1DO8	1.1.1.38	MALIC ENZYME
1FCB	1.1.2.3	FLAVOCYTOCHROME B2
1FNB	1.18.1.2	FERREDOXIN-NADP+ REDUCTASE
1FOH	1.14.13.7	PHENOL HYDROXYLASE
1FVA	1.8.4.11	PEPTIDE METHIONINE SULFOXIDE REDUCTASE
1GAL	1.1.3.4	GLUCOSE OXIDASE
1GCU	1.3.1.24	BILIVERDIN REDUCTASE A
1GDH	1.1.1.29	D-GLYCERATE DEHYDROGENASE
1GOG	1.1.3.9	GALACTOSE OXIDASE

Continued on Next Page…

Supplementary Table 1 – Continued

**PDB**	**EC number**	**Native function**
1GOX	1.1.3.15	(S)-2-HYDROXY-ACID OXIDASE, PEROXISOMAL
1IPH	1.11.1.6	CATALASE HPII
1KNP	1.4.3.16	L-aspartate oxidase
1L1L	1.17.4.2	RIBONUCLEOSIDE TRIPHOSPHATE REDUCTASE
1L6P	1.8.1.8	Thiol:disulfide interchange protein dsbD
1L7D	1.6.1.2	nicotinamide nucleotide Transhydrogenase, sub
1LDM	1.1.1.27	M4 LACTATE DEHYDROGENASE
1LUC	1.14.14.3	BACTERIAL LUCIFERASE
1MBB	1.1.1.158	URIDINE DIPHOSPHO-N-ACETYLENOLPYRUVYLGLUCOSAM
1MPY	1.13.11.2	CATECHOL 2,3-DIOXYGENASE
1NAA	1.1.99.18	Cellobiose dehydrogenase
1POW	1.2.3.3	PYRUVATE OXIDASE
1TDE	1.8.1.9	THIOREDOXIN REDUCTASE
1YBV	1.1.1.252	TRIHYDROXYNAPHTHALENE REDUCTASE
1YLU	1.5.1.34	Oxygen-insensitive NAD(P)H nitroreductase
2ACU	1.1.1.21	ALDOSE REDUCTASE
2CND	1.7.1.1	NADH-DEPENDENT NITRATE REDUCTASE
2ESD	1.2.1.9	NADP-dependent glyceraldehyde-3-phosphate deh
2NAC	1.2.1.2	NAD-DEPENDENT FORMATE DEHYDROGENASE
2PGD	1.1.1.44	6-PHOSPHOGLUCONATE DEHYDROGENASE
2TMD	1.5.8.2	TRIMETHYLAMINE DEHYDROGENASE
3R1R	1.17.4.1	RIBONUCLEOTIDE REDUCTASE R1 PROTEIN
4MDH	1.1.1.37	CYTOPLASMIC MALATE DEHYDROGENASE
5COX	1.14.99.1	CYCLOOXYGENASE-2
