# Supplementary material for: A Measure of the Promiscuity of Proteins and Characteristics of Residues in the Vicinity of the Catalytic Site That Regulate Promiscuity
Source: PLoS One. 2012 Feb 16;7(2):e32011. doi: 10.1371/journal.pone.0032011 (PMC3281107; doi:10.1371/journal.pone.0032011)
Supplement: Table S2 — Proteins added to the CSA list to include some proteins of interest. (PDF) [file pone.0032011.s003.pdf]

Supplementary Table 2: Proteins added to the CSA list to include some proteins of interest

**PDB**	**EC number**	**Native function**
1PO5	1.14.14.1	Cytochrome P450 2B4
1GUM	2.5.1.18	PROTEIN (GLUTATHIONE TRANSFERASE A4-4)
1DBT	4.1.1.23	OROTIDINE 5'-PHOSPHATE DECARBOXYLASE
2GVW	3.1.8.2	Phosphotriesterase
1ONE	4.2.1.11	ENOLASE
1V04	3.1.1.2	SERUM PARAOXONASE/ARYLESTERASE 1
1DPT	4.1.1.84	D-DOPACHROME TAUTOMERASE
1QRG	4.2.1.1	CARBONIC ANHYDRASE
1HDH	3.1.6.1	ARYLSULFATASE
1FJO	3.4.24.27	THERMOLYSIN
1ZNB	3.5.2.6	METALLO-BETA-LACTAMASE
